# Multicolor Super-Resolution Fluorescence Imaging via Multi-Parameter Fluorophore Detection

**DOI:** 10.1002/cphc.201100735

**Published:** 2011-12-23

**Authors:** Mark Bates, Graham T Dempsey, Kok Hao Chen, Xiaowei Zhuang

**Affiliations:** a School of Engineering and Applied Sciences, Graduate program in Biophysics, Department of Chemistry and Chemical Biology, Department of Physics, Howard Hughes Medical Institute, Harvard UniversityCambridge, Massachusetts, 02138 (USA), Fax: (+1) (617) 496-9559 E-mail: zhuang@chemistry.harvard.edu

**Keywords:** fluorescence microscopy, fluorescent probes, multicolor imaging, photoswitchable fluorophores, super-resolution imaging

## Abstract

Understanding the complexity of the cellular environment will benefit from the ability to unambiguously resolve multiple cellular components, simultaneously and with nanometer-scale spatial resolution. Multicolor super-resolution fluorescence microscopy techniques have been developed to achieve this goal, yet challenges remain in terms of the number of targets that can be simultaneously imaged and the crosstalk between color channels. Herein, we demonstrate multicolor stochastic optical reconstruction microscopy (STORM) based on a multi-parameter detection strategy, which uses both the fluorescence activation wavelength and the emission color to discriminate between photo-activatable fluorescent probes. First, we obtained two-color super-resolution images using the near-infrared cyanine dye Alexa 750 in conjunction with a red cyanine dye Alexa 647, and quantified color crosstalk levels and image registration accuracy. Combinatorial pairing of these two switchable dyes with fluorophores which enhance photo-activation enabled multi-parameter detection of six different probes. Using this approach, we obtained six-color super-resolution fluorescence images of a model sample. The combination of multiple fluorescence detection parameters for improved fluorophore discrimination promises to substantially enhance our ability to visualize multiple cellular targets with sub-diffraction-limit resolution.

## 1. Introduction

Fluorescence microscopy is a widely used imaging tool for cell and molecular biology, and one of the strengths of this technique is the variety of fluorescent probes which may be used to label the specimen. Synthetic dye molecules, fluorescent proteins, and inorganic fluorescent nanoparticles are available with a range of spectral properties and may be linked to their target with high specificity through numerous coupling strategies.^1^ In particular, multicolor fluorescence imaging of fluorophores with distinct spectral properties allows for the detection of multiple targets, enabling the observation of interactions and relative spatial organization between different cellular structures with molecule-specific contrast.

The recent development of far-field super-resolution fluorescence imaging techniques,^2^–^7^ with spatial resolution reaching the nanometer scale, has created new opportunities for the study of biological ultrastructure. In general, these methods rely on a number of photophysical or photochemical mechanisms by which fluorescent probes may be converted between a detectable “on” state and a non-detectable “off” state, either at spatially well-defined regions of the sample^2^, ^5^ or in a stochastic manner through the detection of photo-switchable single molecules.^4^, ^6^, ^7^ As compared to conventional imaging, super-resolution fluorescence microscopy places higher demands on the fluorescent probes in terms of brightness, photostability, and in some cases the requirement for photo-switchable fluorescence emission. Such constraints have spurred the identification, development, and characterization of new probes for super-resolution imaging^6^–^11^ and have also led to innovative strategies for obtaining multicolor data. Multicolor super-resolution fluorescence microscopy has been demonstrated by several means, such as employing fluorophores with different fluorescence activation wavelengths,^12^–^15^ fluorophores with well-separated emission spectra,^9^, ^16^–^19^ by ratio-metric imaging of fluorophores with overlapping emission spectra,^9^, ^20^, ^21^ or by taking advantage of other spectral properties, such as fluorescence lifetime.^22^

Stochastic optical reconstruction microscopy (STORM) is a super-resolution fluorescence imaging technique based on sequential, nanometer-scale localization of individual photo-switchable fluorescent labels, which emit light at different times during image acquisition.^23^–^25^ This technique is applicable to any fluorescent dye or protein, which may be switched between distinct spectral states, and in particular between a state that emits fluorescence in a specific wavelength range and a state that does not emit in that range. Among the organic dyes there are numerous examples of photo-activatable, photo-switchable, and blinking fluorophores, emitting over a wide spectral range, which have been demonstrated for use with this approach.^12^, ^21^, ^23^, ^26^–^32^ Particularly, several red-emitting cyanine fluorophores (e.g. Cy5, Alexa 647, Cy5.5, Cy7, and Alexa 750) have been reported to exhibit photo-switchable fluorescence emission.^12^, ^21^, ^23^, ^33^, ^34^ Upon illumination with red excitation light, these dyes emit fluorescence before switching to a non-fluorescent state by forming a thiol adduct. Exposure to UV light, however, removes the thiol group and returns the dye into its fluorescent state.^35^ Interestingly, the reactivation wavelength can be adjusted by pairing a photo-switchable reporter fluorophore (e.g. Alexa 647) with a second fluorophore, termed the activator, having the desired absorption spectrum.^12^ Selective activation of dye pairs with different activation wavelengths has proven to be a robust technique for multicolor super-resolution fluorescence imaging.^12^–^15^

We previously proposed a multi-parameter detection method for multicolor STORM, based on the discrimination of photo-switchable fluorescent probes by both activation wavelength and emission spectrum.^12^ However, this approach has yet to be realized experimentally. Herein we provide the first experimental demonstration of multi-parameter fluorophore detection for multicolor super-resolution imaging. First, we measure the blinking kinetics and brightness of two spectrally separate photo-switchable fluorophores, Alexa 750 and Alexa 647 (dye structures and fluorescence spectra are shown in Figure S1 in the Supporting Information), and use these dyes to obtain two-color STORM images of biological samples. Based on these data, we compare the advantages and disadvantages of two strategies for multicolor STORM imaging: 1) the selective activation method, in which color identification is based on activation wavelength, and 2) a scheme employing multiple fluorescence emission channels to differentiate probes based on their emission spectra. Finally, combining these two approaches, we demonstrate multi-parameter fluorescence detection for super-resolution microscopy, enabling simultaneous imaging of six distinct fluorescent labels within a model sample, with nanoscale spatial resolution.

## 2. Results and Discussion

### Photo-Switching Characteristics of Alexa 750 and Alexa 647

**Figure 1:**
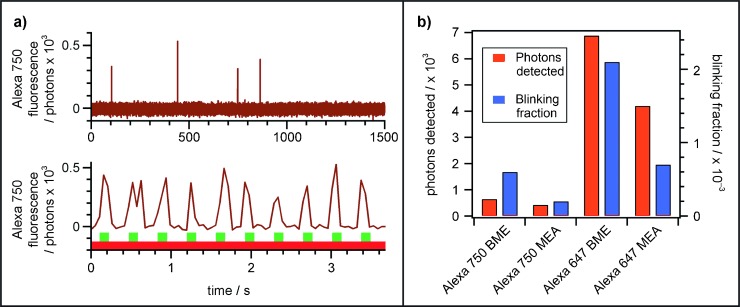
Photoswitching behavior of Alexa 750 and comparison with Alexa 647. a) Top: Blinking of fluorescence emission from Alexa 750 under continuous 752 nm illumination. Bottom: Reversible light-driven switching of the Cy3-Alexa 750 dye pair under continuous 752 nm illumination (indicated by the red bar) and periodic pulses of 532 nm green illumination (indicated by the green bars). The 532 nm light efficiently re-activates Alexa 750 after it is switched off by the 752 nm light. b) Characterization of the number of photons per switching cycle (red bars) and equilibrium blinking fraction (or on-off duty cycle, blue bars), for Alexa 750 in comparison with Alexa 647. Switching characteristics were measured for buffers containing one of two different primary thiols: β-mercaptoethanol (BME) or β-mercaptoethylamine (MEA).

To characterize Alexa 750 fluorescence switching, antibodies were labeled with Alexa 750 and bound non-specifically to a glass coverslip at low density such that individual molecules were resolvable from each other. Alexa 750 was excited at a wavelength close to its absorption peak by using 752 nm light. In the presence of thiol, that is, with β-mercaptoethanol (BME) or β-mercaptoethylamine (MEA) in the imaging buffer, the 752 nm light excites fluorescence from Alexa 750 and also rapidly switches the molecules to a non-fluorescent state. Additionally, 752 nm excitation light can also cause the dye to reactivate back into the fluorescent state, albeit at a much lower rate. Hence, single-molecule Alexa 750 traces exhibited a blinking behavior with sporadic fluorescence bursts (Figure 1 a). Illumination with violet (405 nm) light strongly increased the activation rate (data not shown). To demonstrate that the reactivation wavelength for Alexa 750 can be adjusted into the visible range by pairing it with an activator fluorophore having the appropriate absorption spectrum, we also labeled antibodies with both Alexa 750 and Cy3. Single molecule traces of Cy3-Alexa 750-labeled antibodies, when detected at the Alexa 750 emission wavelength, exhibited a light-driven switching behavior. The 752 nm imaging laser rapidly switched Alexa 750 into a stable dark state that did not emit detectable fluorescence, whereas a brief exposure to low intensity green activation light (532 nm) caused efficient reactivation of the Alexa 750 fluorescence. Both the spontaneous photoswitching of Alexa 750 alone and the Cy3-assisted photoactivation may be used for STORM imaging, as we demonstrate below.

Two properties of photo-switchable fluorophores are particularly important for applications in STORM imaging: 1) the fluorophore brightness, and 2) the equilibrium blinking fraction. The first property, fluorophore brightness, may be quantified in terms of the number of photons detected per switching cycle from single fluorophores. Fluorophores emitting a higher number of photons per cycle are easier to detect and their positions may be determined with a higher degree of precision,^36^, ^37^ leading to a higher spatial resolution in the final image. The second property, the equilibrium blinking fraction (also known as the on-off duty cycle, denoted *ν*) is defined as the fraction of time the dye spends in the fluorescent state under constant illumination with imaging light.^4^ For switchable cyanine dyes, the blinking fraction is largely independent of the excitation light intensity (data not shown). At equilibrium, the number of activated fluorophores per unit volume equals the fluorophore density (*ρ*) times the blinking fraction (*ν*). When *ρν* approaches one per diffraction-limited volume, the images of individual activated fluorophores begin to overlap substantially, thus inhibiting their precise localization. In this manner the equilibrium blinking fraction sets an upper bound for the density of fluorophores which may be present on the sample before blinking interferes with STORM data acquisition.^4^, ^38^, ^39^ High label density is an important prerequisite for high-resolution fluorescence imaging, since closely spaced fluorophores are required in order to map out the fine structural features of a sample. This observation may be restated quantitatively in terms of the Nyquist sampling theorem,^19^, ^40^, ^41^ and hence the steady state blinking fraction of a fluorophore is also a key parameter which affects the overall resolution of a STORM image.^4^

To measure the photon yield and blinking fraction for Alexa 750, we exposed the dye-labeled antibodies to 752 nm light and recorded time traces of their fluorescence emission. The photon yield and blinking fraction of Alexa 750 is plotted in Figure 1 b, in comparison with the same properties measured for Alexa 647 using a similar approach (with the exception that 647 nm illumination was used for Alexa 647). For these measurements antibodies were labeled with the reporter dye (Alexa 750 or Alexa 647) only. Exponential fits to the photon distributions yielded a value of ∼650 photons detected per switching cycle for Alexa 750 and ∼6900 photons for Alexa 647 in an imaging buffer containing BME, and ∼430 photons for Alexa 750 and ∼4200 photons for Alexa 647 in a buffer containing MEA (Figure 1 b and Figure S2 in the Supporting Information). Although not as bright as Alexa 647, the photon yield of Alexa 750 is comparable to that measured for other photo-switchable fluorescent probes used for super-resolution microscopy,^4^, ^6^, ^7^ and single molecules of Alexa 750 are easily detected above background.

The mean steady-state blinking fraction for Alexa 750 is 0.6×10^−3^ in BME buffer and 0.2×10^−3^ in MEA buffer, significantly lower than that of Alexa 647 (Figure 1 b and Figure S3 in the Supporting Information). The blinking fraction was analyzed on a molecule-by-molecule basis, revealing a broad distribution of blinking rates for individual dyes (Figure S3 in the Supporting Information). Overall, the blinking fraction of Alexa 750 indicates that this fluorophore can be switched off effectively by excitation with 752 nm light to an extent such that, on average, only ∼1 molecule in 2000 remains fluorescent at steady state. In contrast, Alexa 647 exhibits a moderately higher rate of blinking for either of the two buffer conditions tested. We found that the photo-switching of Alexa 750 in buffer containing MEA was not robust, with a large fraction of fluorophores photo-bleaching after a small number of switching cycles, and hence this imaging buffer was not used for Alexa 750 STORM imaging. We note that the photo-switching properties of Alexa 647 and Alexa 750 are similar to those of their structural analogues, Cy5 and Cy7 (data not shown).

### Two-Color STORM by Distinguishing Different Reporters or Different Activators

**Figure 2:**
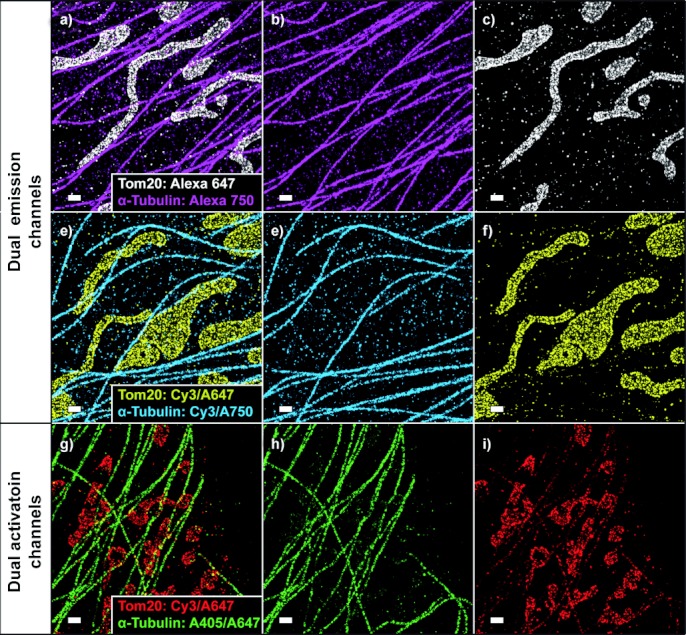
Comparison of two-color STORM images taken with the dual-emission channel scheme and the dual-activation channel scheme. a–c) Dual-emission channel STORM imaging of microtubules and mitochondria in BSC-1 cells immuno-stained with Alexa 750 and Alexa 647, respectively (no activator fluorophore was used). d–f) Dual-emission channel STORM imaging of microtubules and mitochondria in BSC-1 cells immuno-stained with the Cy3-Alexa 750 dye pair and the Cy3-Alexa 647 dye pair, respectively. g–i) Dual-activation channel STORM imaging of microtubules and mitochondria in BSC-1 cells immuno-stained with the Alexa 405-Alexa 647 pair and the Cy3-Alexa 647 pair, respectively. The STORM data shown in panels g–i have been corrected for color crosstalk (see Supporting Information). Scale bars 500 nm.

The emission peaks of Alexa 750 and Alexa 647 are separated by ∼100 nm in wavelength, allowing each dye to be detected independently (Figure S1 in the Supporting Information). Hence, a detection setup using two non-overlapping emission channels is suitable for two-color STORM imaging with these dyes. We obtained dual-emission channel STORM images for mammalian cells fixed and immuno-stained with primary antibodies against α-tubulin and the mitochondrial outer membrane protein Tom20. The secondary antibodies were labeled with Alexa 647 for Tom20 and Alexa 750 for tubulin (without an activator fluorophore), the sample was imaged in BME buffer, and a STORM data set for each emission channel was acquired by stochastically activating and imaging sparse subsets of fluorescent labels and iterating this process over many cycles.^23^–^25^ This data was analyzed to determine the position of each detected fluorophore with nanometer-scale precision, and the final STORM image was generated by plotting the fluorophore positions (Figure 2 a). The images for each channel were co-aligned to within a precision of approximately 5 nm using fiducial marks visible in both channels (Figure S4 in the Supporting Information). We also obtained dual-emission channel images of mitochondria and microtubules using secondary antibodies labeled with the activator-reporter pairs Cy3-Alexa 647 for Tom20 and Cy3-Alexa 750 for tubulin. These two-color STORM images are shown in Figures 2 d–f. The presence of the activator dye in conjunction with the photo-switchable emitter enabled tuning of the overall activation rate with a low-intensity visible activation light source, reducing potential photodamage.^12^, ^33^ These images reveal structural features of the microtubule and mitochondrial organization at length scales well below the diffraction limit, which would be fully obscured in conventional fluorescence imaging (Figure S5 in the Supporting Information).

For purposes of comparison, we obtained two-color STORM images of the same cellular targets using activator-reporter dye pairs with different activators, which were distinguished by their different activation wavelengths, as demonstrated previously.^12^ In this experiment the secondary antibodies were labeled with the Alexa 405-Alexa 647 dye pair for tubulin and the Cy3-Alexa 647 dye pair for Tom20, and the sample was imaged in MEA buffer to minimize dye blinking and color crosstalk (as described below). The dual-activation channel STORM image is shown in Figure 2 g, and the localizations assigned to each color channel are shown in Figures 2 h and i. The correct registration of each color channel is assured for this imaging scheme, and no additional image alignment was necessary since all data was collected using a single detection channel. As in the case of dual-emission channel STORM, the detailed view of the microtubules and mitochondria in Figure 2 g reveals spatial organization at sub-diffraction-limit length scales (Figure S6 in the Supporting Information).

Since both of the photo-switchable dyes used herein may be switched for multiple cycles, it is possible to directly quantify the localization precision from the experimental data by analyzing the distribution of positions obtained for repeated localizations of a single fluorophore within the sample. Throughout the data shown in Figure 2, isolated clusters of localizations are found which may correspond to individual fluorophores on isolated, nonspecifically-bound antibodies. By calculating the standard deviation of the localization distribution for isolated clusters of localizations in the STORM images in Figure 2, we measured the localization precision to be 8 nm for Alexa 647 and 13 nm for Alexa 750. This corresponds to a minimum resolvable separation distance between two fluorophores of ∼19 nm for Alexa 647 and ∼31 nm for Alexa 750 (the full width at half maximum of the distribution; see Figure S7 in the Supporting Information). The lower localization precision obtained for Alexa 750 compared to Alexa 647 was expected, resulting from the lower number of photons obtained for this dye (Figure 1).

### Color Crosstalk in STORM Images

Crosstalk in multicolor fluorescence imaging occurs when the signal from a fluorophore is assigned to the incorrect color channel. The STORM images shown in Figure 2, which were obtained using different methods for color identification, contain varying degrees of color crosstalk arising from different sources. In the case of the dual-emission channel images of Alexa 647 and Alexa 750 (Figures 2 a–f), crosstalk may arise due to leakage of Alexa 647 fluorescence into the Alexa 750 emission channel and vice-versa. Analysis of the fluorescence emission spectra reveals little overlap between the two dyes, however (see Figure S1 in the Supporting Information). As illustrated by the images of the individual color channels shown in Figures 2 b, c, e and f, the crosstalk between the two channels is relatively low, allowing microtubules and mitochondria to be identified unambiguously. A second source of crosstalk arises from non-specific binding by the primary or secondary antibodies, which appears as a background of spots distributed over the image. An analysis of the dual-emission channel STORM images showed that overall, color crosstalk amounts to 6±1 % in the microtubule channel and 5±1 % in the Tom20 channel without crosstalk subtraction, as determined by measuring the number of localizations detected in each channel for regions of the sample containing microtubules only, or mitochondria only (Figure S8 in the Supporting Information).

For the dual-activation channel STORM images (Figures 2 g–i), different mechanisms contribute to color crosstalk. In practice, when taking two-color images with this scheme, the sample is exposed to a sequence of light pulses which activate, excite, and deactivate the fluorophores, and each switching event is assigned to a color based on the wavelength of the preceding activation pulse. Errors in color assignment arise when a fluorophore pair is activated by a pulse of the incorrect wavelength (for example, if the Cy3-Alexa 647 dye pair is activated by 405 nm light). Stochastic dye blinking, independent of the activation light, described above, also contributes to crosstalk, since blinking events that occur during or immediately following an activation pulse are not distinguishable from fluorophores that are specifically activated in response to the activation pulse. Evidence of these sources of crosstalk is visible in Figures 2 g–i. Microtubules are weakly visible in the mitochondrion channel (Figure 2 i) and to a lesser extent mitochondria are visible in the microtubule image (Figure 2 h). The average degree of crosstalk in the images of microtubules and mitochondria was determined to be 19±2 % and 13±1 %, respectively, prior to any crosstalk subtraction (Figure S9 in the Supporting Information).

As is the case for conventional multicolor fluorescence images, crosstalk in multicolor STORM data can be corrected to some extent if the degree of crosstalk between each of the channels is known. Using an established linear unmixing approach,^42^ we applied a statistical crosstalk correction to the dual-activation channel STORM image data, as described previously (see Supporting Information).^12^ In order to calculate the local statistics for each color channel, the STORM image data was coarse grained with a radial bin size of 35 nm. After this procedure, crosstalk in the dual-activation channel images (Figures 2 g–i) was reduced to 10±2 % and 7±1 %, for microtubules and mitochondria respectively (Figure S8 and S9 in the Supporting Information).

The significantly lower degree of crosstalk in the dual-emission channel STORM data, without requiring crosstalk correction, highlights one of the main advantages of multicolor STORM imaging using this scheme with reporters exhibiting a large spectral separation in emission color. Conversely, the dual-activation channel imaging scheme has several advantages in that no channel alignment is required (i.e. registration between different color channels is perfect), chromatic aberration does not affect the image since only one fluorophore is detected, and the brighter photo-switchable reporter molecule (Alexa 647), which yields substantially higher resolution, can be used to image both targets.

### Multi-Parameter Fluorescence Detection

**Figure 3:**
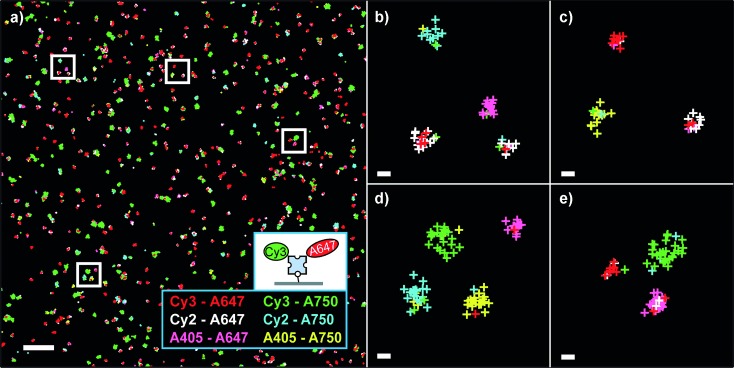
Six-color STORM imaging using multi-parameter detection. a) Six-color STORM image of streptavidin molecules on glass. Streptavidin was dual-labeled with one of three activator fluorophores (Cy3, Cy2, or Alexa 405) and a photo-switchable reporter fluorophore (Alexa 647 or Alexa 750), and bound to a glass coverslip coated with biotin (inset). Each fluorophore localization event was color coded according to the wavelength of the activation pulse which preceded it and the fluorescence channel in which it was detected. Scale bar 500 nm. b–e) Detailed views of the boxed regions in (a) showing the localizations plotted as crosses. Tightly clustered groups of localizations were predominantly composed of a single color, revealing the labeling of each streptavidin molecule. Scale bars 25 nm.

The two methods for discrimination of photo-switchable fluorophores described above, namely identification by activation wavelength or by emission wavelength, are compatible and may be combined in a multi-parameter measurement to increase the number of simultaneously distinguishable probes in a multicolor STORM image. As an example of multicolor STORM using this multi-parameter detection approach, we obtained six-color super-resolution images of a model sample consisting of fluorophore-labeled streptavidin bound to a glass surface. The use of a model sample simplified the demonstration of the method, while avoiding the complications associated with the preparation of a cell sample specifically stained for six distinct targets. Streptavidin molecules were labeled with one of the following six dye pairs: Cy3-Alexa 647, Cy2-Alexa 647, Alexa 405-Alexa 647, Cy3-Alexa 750, Cy2-Alexa 750, or Alexa 405-Alexa 750. The labeled streptavidin samples were mixed and bound to a glass coverslip through a biotin linkage at a high density, such that individual molecules were not resolvable by conventional fluorescence microscopy. The resulting STORM image is shown in Figure 3, revealing closely spaced clusters of differently colored localizations corresponding to individual streptavidin molecules localized multiple times. Each localization was color coded according to the wavelength of the activation pulse which preceded it (532 nm, 458 nm, or 405 nm light to excite Cy3, Cy2, or Alexa 405, respectively), and the emission channel in which the fluorophore was detected (Alexa 647 or Alexa 750). Analysis of the localization clusters yielded a measurement of the color crosstalk between the various dye pairs. Within each cluster, approximately 80–90 % of the localizations were of a single color, thereby identifying the dye pair bound to the streptavidin (Figure 3 and Figure S10 in the Supporting Information). The remaining 10–20 % consisted of the total crosstalk from other channels. The majority of the crosstalk occurred between different activation channels (Figure S10 in the Supporting Information). Crosstalk between the Alexa 647 and the Alexa 750 channels, however, was less than 1 % in all cases, as expected due to the low degree of spectral overlap between the dyes. Numerous streptavidin molecules separated by 50 nm or less were clearly resolved in the STORM image, illustrating sub-diffraction-limit image resolution. This data demonstrates an experimental proof of concept for super-resolution fluorescence imaging with up to six colors. Although the preparation of biological samples specifically labeled with six fluorescent probes is practically challenging (e.g. through immuno-staining), we expect that advances in labeling technology will lead to increased labeling specificity and facilitate simultaneous imaging of multiple targets.^8^, ^43^–^45^

## 3. Conclusions

In summary, we applied multi-parameter fluorescence detection to expand the color palette of fluorescent probes available for super-resolution imaging applications. Using Alexa 750 in conjunction with Alexa 647, we obtained two-color STORM images with multiple emission channels. Compared to imaging with multiple activation channels, the use of two or more fluorescence detection channels for STORM offers a means of obtaining multicolor images with reduced color crosstalk, an important factor in cases where a small number of molecules of one component must be detected in the presence of a high concentration of molecules of a second component. The lower degree of crosstalk in the dual-emission channel scheme is due to the well separated spectra of the two dyes. The crosstalk between the Alexa 647 channel and the Alexa 750 channel was measured to be approximately 1 % meaning that, potentially, as few as one target molecule of a given cellular component could be unambiguously distinguished against a background of 100 molecules of a second labeled component. However, the multi-activation channel imaging scheme also has advantages over the multi-emission channel scheme, particularly in avoiding imaging artifacts due to chromatic aberration or errors in channel alignment, and allows high resolution in all color channels by pairing a bright reporter such as Alexa 647 with different activators.

Finally, we demonstrated six-color super-resolution imaging using multi-parameter fluorescence detection. This approach substantially extends the number of simultaneously detectable probes, and may also be used to improve the accuracy of fluorophore identification. The multi-parameter detection concept may be generalized to any set of orthogonal spectral parameters, and for the implementation reported here we distinguished fluorescent probes by the fluorescence emission and the photo-activation spectra of the probes. The number of colors could be further increased by the addition of activation or emission channels, or by adding other detection parameters such as fluorescence lifetime.^20^, ^22^, ^46^ We anticipate that multicolor super-resolution imaging with this approach could find applications in elucidating the composition and organization of multi-component protein complexes, for example. Super-resolution fluorescence imaging also relies on methods for targeting synthetic dyes and fluorescent proteins to the sample with high specificity, in both living and fixed specimens, and the continued development of fluorophores and fluorescent labeling strategies will enable researchers to fully realize the potential of multicolor biological imaging at the nanoscale.

## Experimental Section

### Preparation of Fluorescently Labeled Probes

Donkey anti-rat antibodies (Jackson ImmunoResearch #712-005-153) and donkey anti-rabbit antibodies (Jackson ImmunoResearch 711-005-152) were labeled with one or two amine-reactive fluorophores. Each antibody was labeled with one fluorophore capable of photo-switchable fluorescence emission (the reporter fluorophore), and some were also labeled with an additional fluorophore (the activator fluorophore) which served to enhance the wavelength-dependent reactivation of the reporter fluorophore. Streptavidin (Invitrogen S-888) was labeled in a similar manner. The labeled probes are summarized in Table S1 in the Supporting Information.

Amine reactive fluorophores were obtained from GE Healthcare (Cy3, Cy2) or Invitrogen (Alexa Fluor 647, Alexa Fluor 750, Alexa Fluor 405). Dye labeling was carried out according to the manufacturer’s instructions, and has been described in detail previously.^47^, ^48^ Briefly, unlabeled antibodies or streptavidin were mixed with one or two amine reactive fluorophores in a sodium bicarbonate buffer (0.1 m, pH 8.5), and the labeling reaction was left to proceed at room temperature for 30 min. The labeled product was separated from unreacted dye by running the reaction mixture over a gel filtration column (Illustra NAP-5 column, GE Healthcare), and eluting in PBS. The labeled product was stored at 4 °C in PBS. Labeling of proteins with two fluorophores was completed in a single reaction step, by adding the reactive dye molecules together into the reaction mixture. A detailed labeling protocol is given in the Supporting Information.

The degree of labeling of the antibodies was measured using a UV/Vis spectrophotometer. The labeling ratio was adjusted by varying the amount of each dye that was added. The average degree of labeling for the activator fluorophores (Cy3, Cy2, or Alexa Fluor 405) was approximately 3.0 fluorophores per antibody or streptavidin molecule, and the average degree of labeling for Alexa Fluor 647 was approximately 0.7 fluorophores per antibody or streptavidin molecule, as described previously.^12^ For Alexa Fluor 750, we found that the highest image quality in STORM experiments was obtained for antibodies with an average degree of labeling of 3.0 dyes per antibody molecule. We did not find this higher degree of labeling to adversely affect the properties of Alexa 750 relevant to STORM imaging.

### STORM Microscope

The microscope used for STORM imaging and single molecule fluorophore characterization has been described in detail previously.^47^ To summarize, an inverted fluorescence microscope (Olympus IX71) was fitted with a 100X oil-immersion objective lens (Olympus, UPLANSAPO100XO) which enabled efficient detection of single fluorophores. A custom-built focus lock system based on the reflection of an infra-red laser from the sample was used to maintain sample focus during all measurements. Either a 100 W mercury lamp with standard fluorescence filter sets (Chroma) or weak 647 nm or 752 nm excitation was used to obtain conventional wide-field fluorescence images of the samples prior to STORM image acquisition. For STORM imaging, photo-switchable Alexa 647 or Alexa 750 were excited using 647 nm light or 752 nm light, respectively. For STORM experiments in which no activator dye was used, the sample was exposed to 405 nm light to increase the activation rates of these fluorophores. For STORM imaging using the activator-reporter pairs, reactivation of probes with Alexa 405, Cy2, or Cy3 as the activator fluorophore was accomplished by illumination with light at a wavelength of 405 nm, 458 nm, or 532 nm, respectively. Alternatively, for measurements in which the 532 nm light source was employed to illuminate fiducial markers (see below), a 568 nm light source was used for activation of probes with Cy3 as the activator. A solid-state diode laser (Coherent, CUBE 405) was used to generate 405 nm light, and a diode pumped solid-state laser (Crystalaser) was used to generate 532 nm light. Laser light at 647 nm, 568 nm, and 458 nm was generated using a mixed gas Ar-Kr laser (Coherent, Innova I70 Spectrum). Laser light at 752 nm was also generated using an Ar-Kr laser (Coherent, Innova I300C). The laser illumination was configured such that the illumination angle could be varied between an epi-illumination geometry and a total internal reflection (TIRF) illumination mode. For STORM data acquisition, the sample was illuminated with oblique illumination (not TIRF) for reduced background signal.^49^, ^50^ Fluorescence detection of Alexa 647 was done using a dichroic mirror with extended reflection (Chroma, Z660DCXRU) and a bandpass emission filter (Chroma, ET700/75). Fluorescence detection of Alexa 750 also used a dichroic mirror with extended reflection (Chroma, Q770DCXR) and a bandpass emission filter (HQ800/60). Fluorescence was detected using an EMCCD camera (Andor Technology, Ixon DU897). The overall magnification of the microscope was 100X, corresponding to a sample area of 160 nm×160 nm imaged onto each pixel of the camera.

### Imaging Buffer

Cyanine dyes exhibit thiol dependent photo-switchable fluorescence, meaning that a thiol is required in solution in order for switching to occur.^33^–^35^ The choice of thiol affects switching kinetics, and we characterized switching for two different thiols in this report: β-mercaptoethanol (BME) and β-mercaptoethylamine (MEA). All single molecule photo-switching characterization experiments and STORM experiments were carried out in an imaging buffer. The imaging buffer consisted of a buffer system with an enzymatic oxygen scavenging system containing glucose, glucose oxidase, and catalase to reduce photobleaching, and the thiol to facilitate photoswitching. The specific compositions of the two buffers are given below.

BME imaging buffer:

Tris (50 mm, pH 8.0)

Sodium chloride (10 mm)

Glucose (10 % w/v)

β-mercaptoethanol (143 mm, Sigma, M3148)

Enzymatic oxygen scavenger system (1 % v/v)

MEA imaging buffer:

Tris (50 mm, pH 8.0)

Sodium chloride (10 mm)

Glucose (10 % w/v)

β-mercaptoethylamine, pH 8.5 (10 mm, Sigma, 30070)

Enzymatic oxygen scavenger system (1 % v/v)

The enzymatic oxygen scavenging system was added to the buffer immediately before use, and the stock solution was prepared by mixing glucose oxidase powder (10 mg, Sigma, G2133) with catalase (50 μL, 20 mg mL^−1^, Roche Applied Science, 106810) in PBS (200 μL), and centrifuging the mixture at 13 000 rpm for 1 min.

### Fluorophore Characterization at the Single-Molecule Level

To characterize the switchable fluorescence emission of Alexa 647 and Alexa 750, fluorescently labeled antibodies were bound to a glass coverslip at low density, such that individual molecules could be observed. Antibodies were bound non-specifically, and observed with TIRF illumination. For the demonstration of fluorescence switching shown in Figure 1 a, antibodies labeled with Alexa 750 alone, or Cy3 and Alexa 750, were used. The molecules were continuously exposed to the 752 nm laser, and in the case of antibodies labeled with Cy3 and Alexa 750, the 532 nm laser was pulsed periodically to activate the fluorophores. The fluorescence of each molecule was recorded by a camera.

For measurements of the blinking fraction and the number of photons per switching event, antibodies labeled with only Alexa 647 or Alexa 750 were used, and the samples were continuously exposed to the red imaging laser (647 nm for Alexa 647, or 752 nm for Alexa 750). Alexa 647 or Alexa 750 were observed to initially switch off, and then to blink on and off stochastically. The fluorescence-versus-time trace of each molecule in the field of view was determined from the data. The number of photons per switching event was calculated by integrating the measured fluorescence for each event. Histograms of the photon counts were fit with an exponential function to determine the average number of detected photons per cycle for Alexa 647 and Alexa 750 in each buffer condition (Figure S2 in the Supporting Information). The blinking fraction for each molecule was calculated by measuring the fraction of the total trace duration for which the molecule was in the fluorescent state, until up to the final blinking event, such that any time period during which the fluorophore had photobleached was neglected. Histograms of the blinking fraction for each fluorophore in different imaging buffers are shown in Figure S3 in the Supporting Information.

### Immunofluorescence

Green monkey kidney BS-C-1 cells were plated in LabTek II 8-well chambered coverglass (Nunc) at a density of 3×10^4^ cells per well. After 16 to 24 h, they were rinsed with phosphate buffered saline (PBS) and fixed with formaldehyde (3 %) and glutaraldehyde (0.1 %) at room temperature in PBS for 10 min. The fixing step was followed by quenching with sodium borohydride (0.1 %) in PBS for 7 min to reduce the unreacted aldehyde groups and fluorescent products formed during fixation. The sodium borohydride solution was prepared immediately before use to avoid hydrolysis. The fixed sample was permeabilized in a blocking buffer (3 % BSA, 0.5 % Triton X-100 in PBS) for 10 min and stained with both of the primary antibodies against tubulin (rat anti-α-tubulin, Abcam ab6160, 1:100 dilution) and Tom20 (rabbit anti-Tom20, Santa Cruz sc11415, 1:50 dilution) for 30 min in a blocking buffer. The sample was then rinsed with a washing buffer (0.2 % BSA, 0.1 % Triton X-100 in PBS) three times for 10 min each. The corresponding secondary antibodies labeled with photo-switchable probes were added to the sample (2.5 μg mL^−1^ final concentration, diluted in a blocking buffer) and left for 30 min at room temperature. The sample was then washed three times for 10 min each with a washing buffer. As a final step to preserve fluorophore binding, the sample was rinsed with PBS and then post-fixed for 10 min at room temperature with formaldehyde (3 %) and glutaraldehyde (0.1 %), and then stored in PBS at 4 °C before imaging.

### Multicolor STORM Imaging

The imaging procedure for multicolor STORM was slightly different depending on whether or not multiple fluorescence emission channels were used. For experiments using a single emission channel, the dataset was acquired in one step. Dual-emission channel data was acquired in two imaging steps—one for each channel. By exciting only one of the two fluorophores at any given time, leakage of fluorescence from the tail of the Alexa 647 emission spectrum into the Alexa 750 detection channel, and vice-versa, was reduced. Simultaneous imaging of the two channels would be possible by simultaneously exciting both fluorophores using two different wavelength laser lines, and by using a dual-view imaging setup to split the emission from the two fluorophores into two paths. For all experiments using multiple activation wavelengths for multicolor imaging, the appropriate activation light sources were pulsed in sequence to selectively activate different populations of fluorescent probes. For all STORM imaging experiments, samples were prepared in BME imaging buffer, with the exception of the single-emission channel dataset (Figures 2 g–i), for which MEA imaging buffer was used to reduce the blinking rate of Alexa 647 and thereby minimize color crosstalk. A detailed description of the STORM imaging procedures for each experiment is given in the Supporting Information.

### STORM Data Analysis and Image Generation

Data analysis for STORM has been described in detail previously.^48^ Aspects of the data processing, which were developed for multi-emission channel STORM, are related to channel alignment, which is described below. Further details of the STORM image generation are given in the Supporting Information.

### Channel Registration for Dual-Emission Channel STORM

Since the dual-emission channel multicolor STORM data is acquired by using two different optical detection paths, a method for image registration is required, in order to overlay the images and generate the final two-color STORM image. Because the dichroic mirrors and emission filters are different for the two channels, and also due to the difference in the spectral ranges detected, each channel may have small differences in magnification, image rotation, shear, and other aberrations. Furthermore, this alignment procedure must have a high degree of precision in order to avoid compromising the spatial resolution of the multicolor STORM image itself. We used fiducial markers (fluorescent beads, Invitrogen, F8810) which were visible in both detection channels for alignment of the data sets. During data analysis for both datasets, the bead positions were localized with high precision over the course of the experiment, based on their images which were visible in the raw data. As a result, each of the two resulting STORM images contained tightly localized clusters of localizations corresponding to the beads fixed to the sample. The set of localizations collected from the Alexa 647 channel was transformed, using a polynomial warp transform to account for differences in magnification, rotation, shear, etc., and then the images were aligned using a rigid translation, based on the bead positions in the image. Control measurements verified that this procedure resulted in an image-registration precision of 5.6±2.5 nm. Further details of the alignment procedures and control measurements are given in the Supporting Information.

### Statistical Crosstalk Correction Procedure

Color crosstalk in STORM images can be reduced by using a statistical analysis equivalent to linear un-mixing in conventional fluorescence images, as described previously.^12^, ^13^ A detailed description and derivation of the statistical crosstalk correction procedure is given in the Supporting Information.

### Crosstalk Analysis of STORM Images

A quantitative measurement of color crosstalk present in the STORM data is shown in the Supporting Information. For two color images of microtubules and mitochondria, crosstalk is measured by choosing four or five regions of the sample containing only microtubules or Tom20, and measuring the relative numbers of localizations in these regions for each of the two channels. Each region picked contained a minimum of one thousand localizations. The average degree of crosstalk across the image was determined by calculating the mean of the crosstalk values measured for each region. The reported error for the average crosstalk corresponds to the standard error of the mean. For analysis of the STORM image of labeled streptavidin, crosstalk was quantified by picking isolated clusters of localizations which appear in the data, most likely corresponding to single streptavidin molecules. These were measured to determine the number of localizations of each color within the clusters, yielding a value for the degree of crosstalk. Further details of the crosstalk-analysis procedures are given in the Supporting Information.

## Supplementary material

Detailed facts of importance to specialist readers are published as ”Supporting Information”. Such documents are peer-reviewed, but not copy-edited or typeset. They are made available as submitted by the authors.

## References

[1] Giepmans BN, Adams SR, Ellisman MH, Tsien RY (2006). Science.

[2] Hell SW (2003). Nat. Biotechnol.

[3] Hell SW (2007). Science.

[4] Bates M, Huang B, Zhuang X (2008). Curr. Opin. Chem. Biol.

[5] Heintzmann R, Gustafsson MGL (2009). Nat. Photon.

[6] Patterson G, Davidson M, Manley S, Lippincott-Schwartz J (2010). Annu. Rev. Phys. Chem.

[7] Huang B, Babcock H, Zhuang X (2010). Cell.

[8] Fernández-Suárez M, Ting AY (2008). Nat. Rev. Mol. Cell Biol.

[9] Gunewardene MS, Subach FV, Gould TJ, Penoncello GP, Gudheti MV, Verkhusha VV, Hess ST (2011). Biophys. J.

[10] Grotjohann T, Testa I, Leutenegger M, Bock H, Urban NT, Lavoie-Cardinal F, Willig KI, Eggeling C, Jakobs S, Hell SW (2011). Nature.

[11] Brakemann T, Stiel AC, Weber G, Andresen M, Testa I, Grotjohann T, Leutenegger M, Plessmann U, Urlaub H, Eggeling C, Wahl MC, Hell SW, Jakobs S (2011). Nat Biotechnol.

[12] Bates M, Huang B, Dempsey GT, Zhuang X (2007). Science.

[13] Huang B, Jones SA, Brandenburg B, Zhuang X (2008). Nat. Methods.

[14] Wu M, Huang B, Graham M, Raimondi A, Heuser JE, Zhuang X, De Camilli P (2010). Nat. Cell Biol.

[15] Dani A, Huang B, Bergan J, Dulac C, Zhuang X (2010). Neuron.

[16] Donnert G, Keller J, Wurm CA, Rizzoli SO, Westphal V, Schonle A, Jahn R, Jakobs S, Eggeling C, Hell SW (2007). Biophys. J.

[17] Shroff H, Galbraith CG, Galbraith JA, White H, Gillette J, Olenych S, Davidson MW, Betzig E (2007). Proc. Natl. Acad. Sci. USA.

[18] Bock H, Geisler C, Wurm CA, Von Middendorff C, Jakobs S, Schonle A, Egner A, Hell SW, Eggeling C (2007). Appl. Phys. B.

[19] Jones SA, Shim S-H, He J, Zhuang X (2011). Nat. Methods.

[20] Testa I, Wurm CA, Medda R, Rothermel E, von Middendorf C, Fölling J, Jakobs S, Schönle A, Hell SW, Eggeling C (2010). Biophys. J.

[21] Baddeley D, Crossman D, Rossberger S, Cheyne JE, Montgomery JM, Jayasinghe ID, Cremer C, Cannell MB, Soeller C (2011). PLoS One.

[22] Bückers J, Wildanger D, Vicidomini G, Kastrup L, Hell SW (2011). Opt. Express.

[23] Rust MJ, Bates M, Zhuang X (2006). Nat. Methods.

[24] Betzig E, Patterson GH, Sougrat R, Lindwasser OW, Olenych S, Bonifacino JS, Davidson MW, Lippincott-Schwartz J, Hess HF (2006). Science.

[25] Hess ST, Girirajan TP, Mason MD (2006). Biophys. J.

[26] Fölling J, Belov V, Kunetsky R, Medda R, Schonle A, Egner A, Eggeling C, Bossi M, Hell SW (2007). Angew. Chem. Int. Ed.

[27] Heilemann M, van de Linde S, Schüttpelz M, Kasper R, Seefeldt B, Mukherjee A, Tinnefeld P, Sauer M (2008). Angew. Chem. Int. Ed.

[28] Lord SJ, Lee H-lD, Samuel R, Weber R, Liu N, Conley NR, Thompson MA, Twieg RJ, Moerner WE (2010). J. Phys. Chem. B.

[29] Vogelsang J, Cordes T, Forthmann C, Steinhauer C, Tinnefeld P (2009). Proc. Natl. Acad. Sci. USA.

[30] Belov VN, Bossi ML, Fölling J, Boyarskiy VP, Hell SW (2009). Chem. Eur. J.

[31] Heilemann M, van de Linde S, Mukherjee A, Sauer M (2009). Angew. Chem. Int. Ed.

[32] Lee H-lD, Lord SJ, Iwanaga S, Zhan K, Xie H, Williams JC, Wang H, Bowman GR, Goley ED, Shapiro L, Twieg RJ, Rao J, Moerner WE (2010). J. Am. Chem. Soc.

[33] Bates M, Blosser TR, Zhuang X (2005). Phys. Rev. Lett.

[34] Heilemann M, Margeat E, Kasper R, Sauer M, Tinnefeld P (2005). J. Am. Chem. Soc.

[35] Dempsey GT, Bates M, Kowtoniuk WE, Liu DR, Tsien RY, Zhuang X (2009). J. Am. Chem. Soc.

[36] Thompson RE, Larson DR, Webb WW (2002). Biophys. J.

[37] Yildiz A, Forkey JN, McKinney SA, Ha T, Goldman YE, Selvin PR (2003). Science.

[38] Holden SJ, Uphoff S, Kapanidis AN (2011). Nat. Methods.

[39] Huang F, Schwartz SL, Byars JM, Lidke KA (2011). Biomed. Opt. Express.

[40] Shroff H, Galbraith CG, Galbraith JA, Betzig E (2008). Nat. Methods.

[41] Wolter S, Endesfelder U, van de Linde S, Heilemann M, Sauer M (2011). Opt. Express.

[42] Carlsson K, Mossberg K (1992). J. Microsc.

[43] Uttamapinant C, White KA, Baruah H, Thompson S, Fernández-Suárez M, Puthenveetil S, Ting AY (2010). Proc. Natl. Acad. Sci.

[44] Hampel S, Chung P, McKellar CE, Hall D, Looger LL, Simpson JH (2011). Nat. Methods.

[45] López-Colón D, Jiménez E, You M, Gulbakan B, Tan W (2011). Wiley Interdiscip. Rev. Nanomed. Nanobiotechnol.

[46] Widengren J, Kudryavtsev V, Antonik M, Berger S, Gerken M, Seidel CAM (2006). Anal. Chem.

[47] Dempsey GT, Wang W, Zhuang X, Hinterdorfer P, van Oijen AM (2009). Handbook of Single-Molecule Biophysics.

[48] Bates M, Jones SA, Zhuang X, Yuste R (2011). Imaging: A Laboratory Manual.

[49] Cui BX, Wu CB, Chen L, Ramirez A, Bearer EL, Li WP, Mobley WC, Chu S (2007). Proc. Natl. Acad. Sci. USA.

[50] Tokunaga M, Imamoto N, Sakata-Sogawa K (2008). Nat. Methods.

