# Supplementary material for: Multicolor Super-Resolution Fluorescence Imaging via Multi-Parameter Fluorophore Detection
Source: Chemphyschem. 2011 Dec 23;13(1):99–107. doi: 10.1002/cphc.201100735 (PMC3353744; doi:10.1002/cphc.201100735)
Supplement: Supplementary file 1 [file cphc0013-0099-SD1.pdf]

CHEMPHYSCHEM

Supporting Information

© Copyright Wiley-VCH Verlag GmbH & Co. KGaA, 69451 Weinheim, 2011

Multicolor Super-Resolution Fluorescence Imaging through Multi-Parameter Fluorophore Detection

Mark Bates, Graham T. Dempsey, Kok Hao Chen, and Xiaowei Zhuang*[a]

cphc_201100735_sm_miscellaneous_information.pdf

Supporting Information

Table of Contents

Sample protocol for the preparation of photoswitchable labeled antibodies........................2
Multicolor STORM imaging procedures..3
Single emission channel, dual activation channel imaging of tubulin labeled with Alexa 405 – Alexa 647 and Tom20 labeled with Cy3 – Alexa 647...3
Dual emission channel imaging of tubulin labeled with Alexa 750 and Tom20 labeled with Alexa 647...3
Dual emission channel, single activation channel imaging of tubulin labeled with Cy3 – Alexa 750 and Tom20 labeled with Cy3 – Alexa 647...4
Multi-parameter imaging of labeled streptavidin via two fluorescence detection channels and three activation channels...4
STORM data analysis and image generation..4
Channel registration for dual emission channel STORM..5
Statistical crosstalk correction procedure 6
Crosstalk analysis of STORM images 7
Table S1: Fluorescent antibodies or streptavidin...9
Figure S1: Chemical structures and spectral properties of Alexa 647 and Alexa 750 10
Figure S2: Photons detected per switching event for Alexa 647 and Alexa 750................11
Figure S3: Blinking fraction distributions for Alexa 647 and Alexa 750...........................12
Figure S4: Image registration accuracy 13
Figure S5: Image detail in dual emission channel STORM data..14
Figure S6: Image detail in dual activation channel STORM data 15
Figure S7: Localization precision of Alexa 750 and Alexa 647...16
Figure S8: Crosstalk analysis for the dual emission channel STORM data 17
Figure S9: Crosstalk analysis for the dual activation channel STORM data......................18
Figure S10: Crosstalk analysis for the six-color STORM image shown in Figure 3 19
References...20

Sample protocol for the preparation of photoswitchable labeled antibodies:

Many synthetic fluorescent dye molecules exhibit photoswitchable fluorescence emission. In particular, photoswitchable cyanine fluorophores such as Cy5, Alexa 647, Cy7, Alexa 750, etc. may be paired with a second fluorophore which serves as an "activator", determining the wavelength of light that re-activates the fluorescence of the photoswitchable molecule.[1] A protocol for the preparation of antibodies labeled with one such pairing scheme of synthetic fluorophores, Alexa 405 and Alexa 647, is given here. This protocol may easily be adapted for labeling with other fluorophores or for labeling other substrate molecules.

Dye aliquot preparation:

One 1.0 mg tube of Alexa Fluor 405 dye (Invitrogen, A-30000) was dissolved in anhydrous DMSO and divided into 50 aliquots of 0.02 mg each. These were dried and stored at -20C.

One 1.0 mg tube of Alexa Fluor 647 dye (Invitrogen, A-20006) was dissolved in anhydrous DMSO and divided into 50 aliquots of 0.02 mg each. These were dried and stored at -20C.

Antibody labeling:

1. Dissolve the Alexa Fluor 647 dye aliquot in 40 μL of DMSO.
2. Dissolve the Alexa Fluor 405 dye aliquot in 10 μL of DMSO.
3. Add 40 μL secondary antibody (Jackson Immuno Research, donkey anti-mouse, 1.25 mg mL^{-1} stock) to 10 μL $NaHCO_3$ solution (0.5 M, pH ~ 8.5).
4. Add 1.0 μL of Alexa 647 and 5 μL of Alexa 405 solution to the reaction and mix thoroughly.
5. Incubate the reaction for 30 minutes in the dark at RT (rocking gently)
6. Add PBS to bring the final volume of the reaction to 200 μL.
7. Load the reaction onto a size exclusion column (Illustra NAP5, GE Healthcare) pre-equilibrated with PBS.
8. Collect 300 μL fractions in PBS. When using a NAP5 column, the labeled antibody is expected to elute in fraction #3. Verify the peak fraction and the degree of labeling using a UV/Vis spectrophotometer, and store the labeled antibody fraction at 4 degrees C.

These reaction conditions yield a labeling ratio of approximately 3 Alexa 405 dyes per antibody and 0.7 Alexa 647 dyes per antibody (on average) as determined by UV/Vis absorbance measurements. The labeling ratio can be adjusted by varying the amount of each dye that is added. The final concentration of antibody in the recovered fraction should be approximately 0.1 mg mL^{-1}, and this may be used at a dilution of approximately 1:200 for immunofluorescence labeling.

Multicolor STORM imaging procedures

Single emission channel, dual activation channel imaging of tubulin labeled with Alexa 405 – Alexa 647 and Tom20 labeled with Cy3 – Alexa 647

Multicolor STORM imaging using multiple activation wavelengths, in which color discrimination is based on selective activation of different photoswitchable dye pairs, has been described previously.[1] For this set of experiments, imaging was carried out in MEA imaging buffer to reduce the blinking of Alexa 647 and therefore minimize color crosstalk due to stochastic blinking. STORM data was acquired at a camera frame rate of 60 Hz. The sample was exposed to a repeating illumination sequence of 1 frame (17 milliseconds) of 568 nm light, 5 frames (83 milliseconds) of 647 nm light, 1 frame of 405 nm light, and 5 frames of 647 nm light. This sequence served to alternately activate and excite (as well as switch off) the Cy3 – Alexa 647 dye pair and the Alexa 405 – Alexa 647 dye pair. A total of 180000 frames of data were acquired. Single fluorophore switching events which were detected immediately following a 568nm pulse were identified as Cy3 – Alexa 647, and the corresponding localizations in the STORM image were false colored as green in Figure 2G-I. Fluorophore switching events which were detected immediately after a 405 nm pulse were identified as Alexa 405 – Alexa 647, and the corresponding localizations were false colored as red in Figure 2G-I. All fluorescence was detected using a single optical detection path, and therefore no color channel registration was necessary.

Dual emission channel imaging of tubulin labeled with Alexa 750 and Tom20 labeled with Alexa 647

Dual emission channel imaging was carried out in two steps. First, a STORM image was obtained for the Alexa 750 channel, followed by an image of the Alexa 647 channel. Fiducial markers were used for registration of the two images to create the final two-color image. Fluorescent beads (Invitrogen, F8810) bound to the sample were used as fiducial markers for this purpose. Immediately prior to imaging, the sample was incubated with a solution of fluorescent beads (2% solids stock solution diluted 1:50000 in PBS). After allowing the beads to bind for 1 minute, the sample was rinsed and incubated with PBS + 50mM $MgCl_2$, which caused the beads to bind strongly to the surface of the coverglass. Finally, the buffer was exchanged for imaging buffer, and the STORM data was collected. For all experiments involving STORM imaging of Alexa 750, the sample was prepared using BME imaging buffer, since Alexa 750 exhibited a higher rate of permanent photobleaching in MEA buffer. Alexa 647 and Alexa 750 were allowed to blink on and off under the influence of the imaging wavelength (647nm or 752nm for Alexa 647 or Alexa 750, respectively). Localizations determined from individual blinking events were plotted in Figure 2A-C (yellow if the localization is detected in the Alexa 647 channel and cyan if the localization is detected in the Alexa 750 channel) In both cases, a weak 405 nm continuous excitation was used to increase the rate of activation, thereby increasing the total number of localizations in the image. For the Alexa 750 dataset, the camera frame rate was set to 30 Hz and a total of 60000 frames were acquired. For the subsequent Alexa 647 dataset, a camera frame rate of 60 Hz was used and 98000 frames of data were acquired.

Dual emission channel, single activation channel imaging of tubulin labeled with Cy3 – Alexa 750 and Tom20 labeled with Cy3 – Alexa 647

For dual-channel experiments where the Cy3 activator was used (Figure 2D-F), the sample was also imaged sequentially, as described above., except that 568 nm light was used to facilitate activation of these dye pairs. For the Alexa 750 dataset, the camera frame rate was set to 30 Hz, and the sample was exposed to a repeating sequence of 1 frame (50 milliseconds) of 568nm light followed by 4 frames (200 milliseconds) of 752 nm light. The 752 nm illumination served to excite and switch off the Cy3-Alexa 750 dye pairs, and the 568 nm caused re-activation of the Cy3 – Alexa 750 pairs. For the subsequent Alexa 647 dataset, a camera frame rate of 60 Hz was used, and the sample was illuminated with a repeating sequence of 2 frames (33 milliseconds) of 568 nm light followed by 3 frames (50 milliseconds) of 647 nm light. A total of 60000 frames of data were acquired for the Alexa 750 channel, and 120000 frames of data for the Alexa 647 channel.

Multi-parameter imaging of labeled streptavidin via two fluorescence detection channels and three activation channels

The six-color STORM image of labeled streptavidin (Figure 3) was acquired as for the dual-emission channel data described above, with the addition of periodic excitation with three light sources to selectively activate different probes. For this experiment, the BME imaging buffer was used. The data was acquired in two steps, with the Alexa 750 channel imaged first, followed by the Alexa 647 channel. During each imaging step, the sample was exposed to a repeating illumination sequence of one frame 568 nm activation light, 5 frames imaging light (647 nm for Alexa 647, 752 nm for Alexa 750), one frame 458 nm activation light, 5 frames imaging light, 1 frame 405 nm activation light, and 5 frames imaging light. This sequence served to alternately activate and excite (as well as switch off) the reporter dyes Alexa 647 or Alexa 750 which were paired with the activator dyes Cy3, Cy2, or Alexa 405. Localizations were identified and false-colored in the STORM image according to which emission channel they were detected in and which activation pulse immediately preceded their detection. Data for the two emission channels were aligned using fiducial marks as described above.

STORM data analysis and image generation

For all STORM data reported here, a minimum photon threshold of 500 photons was used to screen out localizations with low inherent localization precision. In all dual emission channel experiments, fiducial markers (fluorescent beads bound to the sample) were used for channel alignment by periodically imaging them over the course of data acquisition, and these markers were also used for drift correction. For experiments using a single emission channel, drift correction was accomplished by dividing the STORM dataset into successive time windows and calculating the correlation function between the corresponding STORM images, as described previously.[2] Briefly, by rendering STORM images corresponding to subsets of the data from successive time windows, the sample drift can be calculated from the

data itself. The 2D spatial correlation function between successive images was calculated, and the offset of the centroid of the correlation function provided a direct measure of the sample drift between successive time points. The drift was then subtracted from the position of each localization based on the time point at which the localization was obtained. Density filtering was used to reduce background (spurious localizations) in the streptavidin STORM images. This is equivalent to background subtraction in conventional fluorescence images, and was achieved by filtering out localizations for which the local density of localizations was lower than a given density threshold. The local density of localizations was calculated for each localization in the data set, within an area defined by a circular radius of 35 nm surrounding the localization. For the streptavidin STORM image data shown in Figure 3, a minimum density threshold of 2.5×10^{-3} nm^{-2} was used.

Channel registration for dual emission channel STORM

Fiducial markers (fluorescent beads, Invitrogen, F8810) which were visible in both detection channels were used for alignment of the data sets. The absorption and emission spectra of the beads was sufficiently broad that their fluorescence could be detected in both the Alexa 647 channel and the Alexa 750 channels. In the case of the Alexa 647 channel, exposure to 647 nm light caused the beads to fluoresce and appear in the STORM dataset with brightness similar to single Alexa 647 fluorophores. For the Alexa 750 channel, the beads were visible in the dataset when the 532 nm green laser was pulsed, also appearing with brightness similar to single fluorophores. Therefore, during the collection of dual emission channel STORM data, in addition to any activation light sources, a 532 nm light source was pulsed every 2 seconds in order to excite the fluorescent beads, such that the fiducial markers appeared periodically throughout the data sets. During data analysis for both datasets, the bead positions were localized with high precision over the course of the experiment, based on their images which were visible in the raw data. As a result, each of the two resulting STORM images contained tightly localized clusters of localizations corresponding to the beads fixed to the sample.

Based on images of fixed fluorescent beads which are visible in both channels, a second order polynomial warp transform (POLYWARP procedure, IDL programming language, ITT Visual Information Solutions, Boulder, CO, USA) was used to calculate the overall image transformation between the two channels. Using this image transformation, the set of localizations collected from the Alexa 647 channel, including the bead localizations, were first transformed to the Alexa 750 channel coordinate system. A final rigid translation was then performed, aligning the clusters of bead localizations from the two channels, to precisely overlay the two datasets.

A control measurement was used to determine the accuracy of this alignment procedure. A sample of BSC-1 cells was immunostained for tubulin, as described above, using the rat anti-tubulin antibody, followed by secondary staining with donkey anti-rat antibodies. In this experiment a mixture of two secondary antibodies was used: Donkey anti-rat labeled with Cy2 and Alexa 647, and Donkey anti-rat labeled with Cy2 and Alexa 750. In this manner the same tubulin filaments were labeled with both Alexa 647 and Alexa 750, and

the resulting STORM images of the identical sample in two channels were used to gauge the accuracy of the image alignment. As described above, fluorescent beads were used as fiducial markers.

After image warping and alignment of the clusters of localizations corresponding to the beads, the residual offset between the centers of the bead clusters was measured. The mean residual offset after alignment was 5.6 ± 2.5 nm, which corresponds to the average channel alignment error across the STORM image. Images of the aligned STORM data and a histogram of the residual alignment errors measured for the beads are shown in Figure S4.

Statistical crosstalk correction procedure

Color crosstalk in STORM images can be reduced using a statistical analysis equivalent to linear unmixing in conventional fluorescence images, as described previously.[1, 3] Briefly, the local density of localizations in each color channel is modeled as consisting of a true density which would be observed in the absence of any crosstalk, with an additional contribution of crosstalk from the other color channels. For the two-channel case, we have:

$\mathbf{d_1(r)}$ = true localization density (without crosstalk), channel 1,
$\mathbf{d_2(r)}$ = true localization density (without crosstalk), channel 2,
$\mathbf{D_1(r)}$ = observed localization density, channel 1,
$\mathbf{D_2(r)}$ = observed localization density, channel 2,

and the observed number of localizations in each channel is modeled as follows:

$$D_1(\vec{r}) = d_1(\vec{r}) + C_{21} \times d_2(\vec{r}),$$

$$D_2(\vec{r}) = d_2(\vec{r}) + C_{12} \times d_1(\vec{r}),$$

where C_{12} and C_{21} are coefficients determining the degree of crosstalk in each direction. We can write an expression for $\mathbf{P_{correct}}$, the probability that the color of a certain localization event in a given channel is correctly assigned, as follows:

$$P_{correct,ch1} = \frac{d_1(\vec{r})}{D_1(\vec{r})}.$$

Solving for $\mathbf{P_{correct}}$ in terms of $\mathbf{D_1(r)}$, $\mathbf{D_2(r)}$, $\mathbf{C_{12}(r)}$, and $\mathbf{C_{21}(r)}$ we arrive at

$$P_{correct,ch1} = \frac{1}{1-C_{12}C_{21}}\left(1 - \frac{C_{21}D_2(\vec{r})}{D_1(\vec{r})}\right).$$

Thus, if the crosstalk coefficients are known, the probability that each localization has been assigned the correct color can be calculated based on the observed localization densities in that region of the image. Calculation of the localization densities requires binning over a certain area, and in our calculations we employed a circular area around each localization with a radius of 35 nm. Based on the calculated probabilities, the color of each localization is then retained or switched according to a Monte Carlo algorithm. The crosstalk coefficients may be measured directly from the image, by measuring the numbers of localizations in each

channel for regions of the sample containing only one of the two fluorescent labels (e.g. a region of the image containing only microtubules, or only Tom20). Alternatively, they may be determined by repeating the experiment with only one of the two fluorescent labels added to the sample.

As is the case for conventional linear unmixing approaches, the procedure breaks down when the degree of crosstalk between the channels is very large. Given a particular experimental system with known crosstalk coefficients, it can be shown that the uncertainty in the calculation of $\mathbf{P_{correct}}$ goes as

$$\Delta P_{correct,ch1} \propto \frac{C_{21}}{1-C_{12}C_{21}},$$

and thus the uncertainty grows without bound when the crosstalk coefficients approach unity. This situation may arise in particular when at high fluorophore densities when stochastic fluorophore blinking events constitute a large majority of the recorded switching events. Since the blinking events are randomly distributed between the various activation channels, the effective degree of crosstalk rises and can be difficult or impossible to correct.

Another aspect of the statistical crosstalk correction approach is the requirement for coarse-graining of the localization data by binning, which is done in order to calculate the local densities of localizations. In our analysis the local density of localizations for each channel was calculated at each localization position, accounting for all localizations within a radial distance of 35nm. The minimum size of the binning area is dependent on the number of localizations obtained, and smaller areas could be used. The requirement for binning necessarily reduces the spatial resolution of the crosstalk correction scheme itself, however, as the algorithm cannot correct color crosstalk accurately in regions of the image at which the color changes over length scales shorter than the binning radius. For the data presented in this report, only the dual-activation wavelength STORM images of microtubules and Tom20 (Figure 2G – I) were corrected for crosstalk using this method.

Crosstalk analysis of STORM images

A quantitative measurement of color crosstalk present in the STORM data is shown in Figures S8, S9, and S10. In Figures S8 and S9, crosstalk is measured by choosing regions of the sample containing only microtubules or Tom20, and measuring the relative numbers of localizations in these regions for each of the two channels. Localizations of the incorrect color are present in these regions due to i) color crosstalk mechanisms, ii) non-specific antibody binding, or iii) the presence of tubulin or Tom20 which were not incorporated into microtubules or mitochondria.

For the dual-activation channel STORM data of Figure 2G – I and Figure S9, the regions of the image used to measure the remaining crosstalk were different from those used to determine the crosstalk coefficients. Since this sample contains regions with only one of the two labeled components (i.e. only microtubules, or only Tom20) it is possible to measure the crosstalk coefficients directly from the image data. The resulting crosstalk correction

provides a measure of the limitations of this method therefore, as the residual color crosstalk after correction represents an ideal case for which the crosstalk coefficients are well known. A more general approach is needed when the sample structure is not known a priori, and it cannot be assumed that certain regions of the sample are labeled with only one of the probes. In this scenario the crosstalk coefficients can be measured using singly-labeled samples. Alteratively, a crosstalk correction strategy which removes crosstalk due to stochastic blinking and requires no prior knowledge of crosstalk coefficients may be employed.[4]

For analysis of the STORM image of labeled streptavidin, crosstalk is quantified by picking isolated clusters of localizations which appear in the data, most likely corresponding to single streptavidin molecules. These were measured to determine the number of localizations of each color within the clusters. The clusters were grouped according to which color comprised the majority of localizations within the cluster, and clusters containing less than 10 localizations were excluded from analysis. The overall composition of each group of clusters is shown in Figure S10. For each group, one color constitutes the majority of the localizations and identifies which fluorophores are bound to the streptavidin. The other colors constitute crosstalk, which occurs mainly between the activation channels due to activation by the incorrect wavelength and/or due to stochastic blinking.

Antibody or Streptavidin	Activator fluorophore	Photo-switchable reporter fluorophore
Donkey anti-Rat IgG	none	Alexa Fluor 750
Donkey anti-Rat IgG	Cy3	Alexa Fluor 750
Donkey anti-Rat IgG	Cy3	Alexa Fluor 647
Donkey anti-Rat IgG	Cy2	Alexa Fluor 750
Donkey anti-Rat IgG	Cy2	Alexa Fluor 647
Donkey anti-Rabbit IgG	none	Alexa Fluor 647
Donkey anti-Rabbit IgG	Cy3	Alexa Fluor 647
Donkey anti-Rabbit IgG	Alexa Fluor 405	Alexa Fluor 647
Streptavidin	Cy3	Alexa Fluor 750
Streptavidin	Cy2	Alexa Fluor 750
Streptavidin	Alexa Fluor 405	Alexa Fluor 750
Streptavidin	Cy3	Alexa Fluor 647
Streptavidin	Cy2	Alexa Fluor 647
Streptavidin	Alexa Fluor 405	Alexa Fluor 647

Table S1: Fluorescent antibodies or streptavidin. The fluorescent antibodies or streptavidin molecules used in the experiments are listed here, along with the fluorophore or combination of fluorophores with which they were labeled.

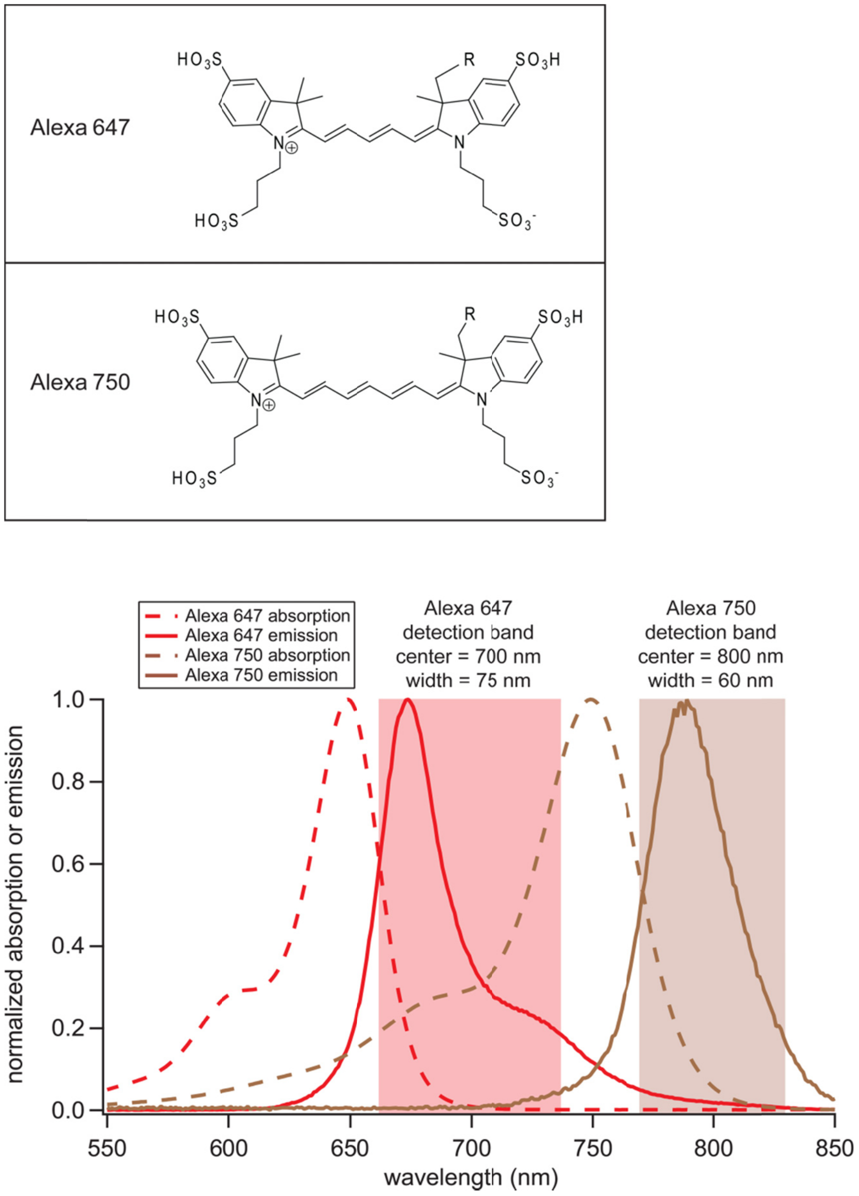

Figure S1: Chemical structures and spectral properties of Alexa 647 and Alexa 750. The upper panels show the chemical structures of Alexa 647 and Alexa 750, where R represents the linker to the reactive group used to couple the dye to the antibody or other molecule.[5] The lower panel shows the absorption and emission spectra of Alexa 647 and Alexa 750. The data have been normalized to the maximum of each curve. The detection bands for Alexa 647 and Alexa 750, as determined by the emission filters, are denoted by the shaded areas on the graph.

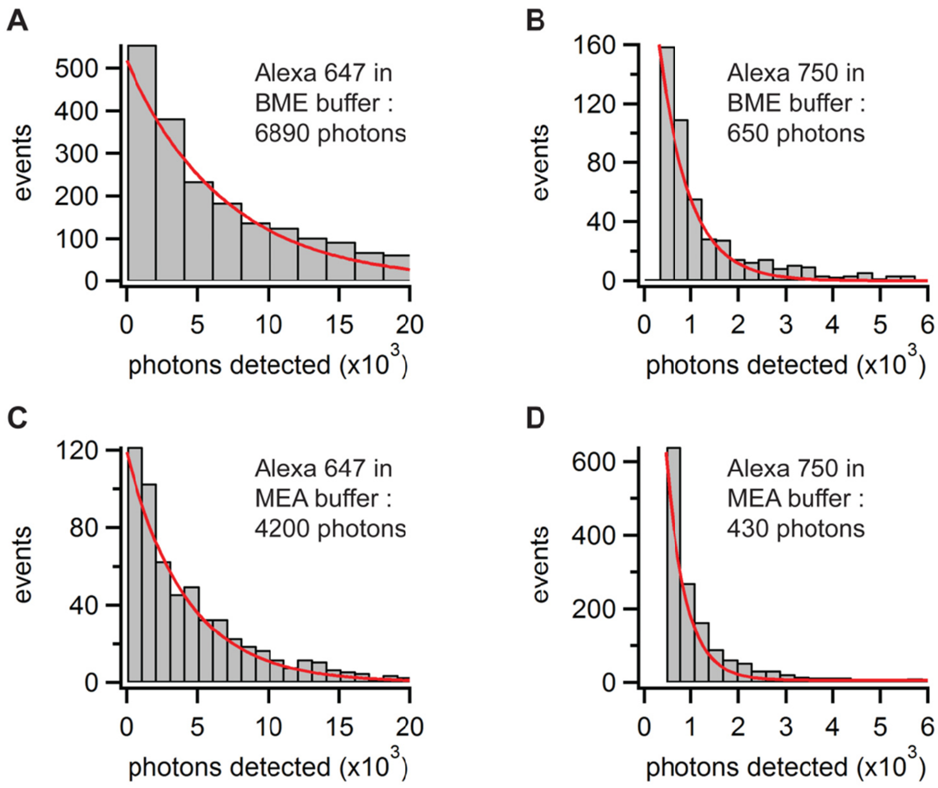

Figure S2: Photons detected per switching event for Alexa 647 and Alexa 750. For each case, the histogram (grey bars) was fit with an exponential decay function of the form $y = \exp(-x / \tau)$ (red line), where τ is the characteristic photon count. (**A**) Alexa 647 switching in BME buffer, characteristic photon count = 6890 photons. (**B**) Alexa 750 switching in BME buffer, characteristic photon count = 650 photons. (**C**) Alexa 647 switching in MEA buffer, characteristic photon count = 4200 photons. (**D**) Alexa 750 switching in MEA buffer, characteristic photon count = 430 photons.

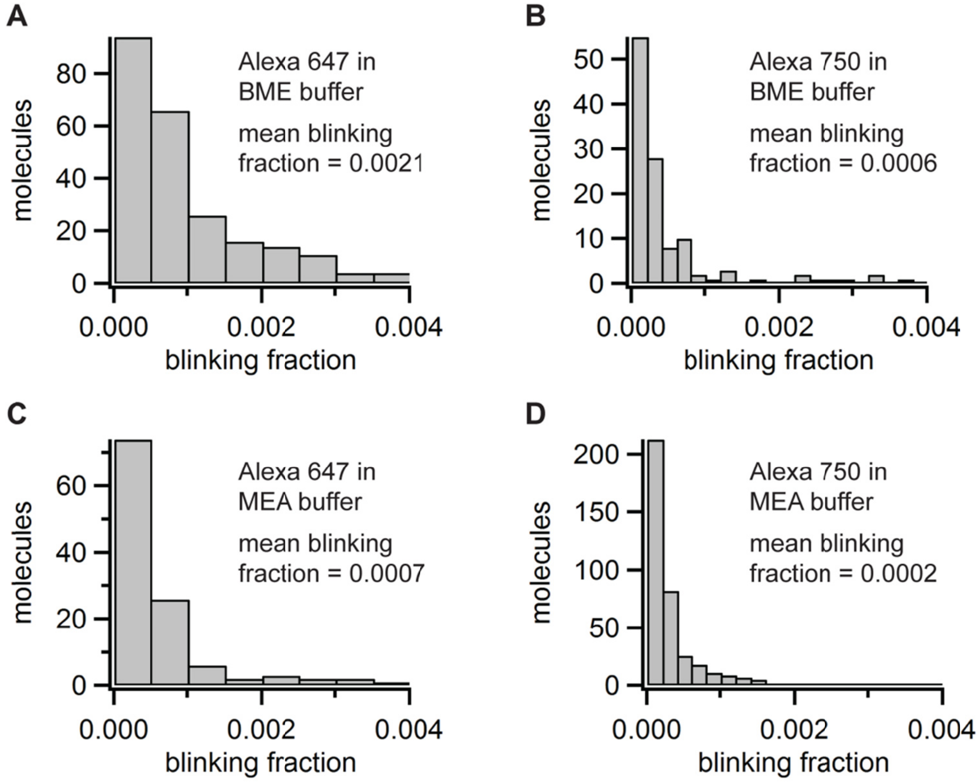

Figure S3: Blinking fraction distributions for Alexa 647 and Alexa 750. Each count in the histogram corresponds to a single-molecule fluorescence time trace. (**A**) Alexa 647 blinking in BME buffer, mean blinking fraction = 0.002. (**B**) Alexa 647 blinking in MEA buffer, mean blinking fraction = 0.0007. (**C**) Alexa 750 blinking in BME buffer, mean blinking fraction = 0.0006. (**D**) Alexa 750 blinking in MEA buffer, mean blinking fraction = 0.0002.

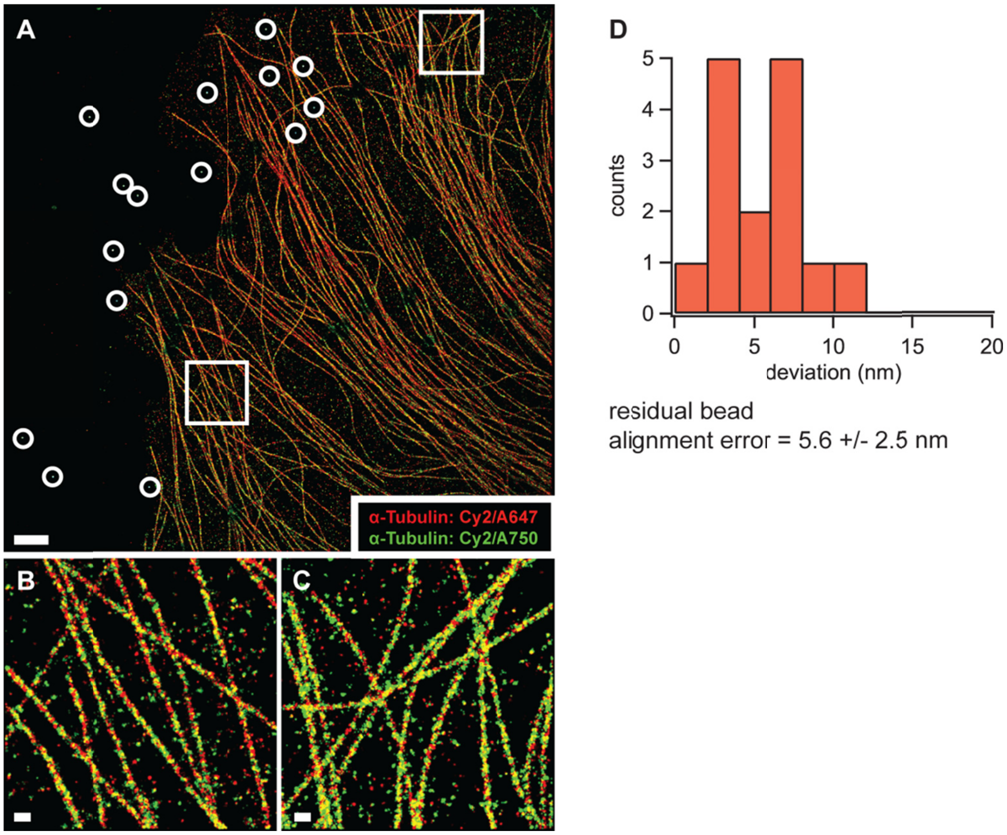

Figure S4: Image registration accuracy. (**A**) A mammalian cell was immunolabeled with rat anti-α-tubulin primary antibody, and donkey anti-rat secondary antibodies. A mixture of two secondary antibodies was used: donkey anti-rat labeled with Cy2 and Alexa 647, and donkey anti-rat labeled with Cy2 and Alexa 750, such that the same tubulin filaments appear in the STORM images of both the Alexa 647 channel (shown in red) and the Alexa 750 channel (shown in green). The positions of the fiducial marks (not visible in the image) are indicated by white circles. Alignment of the Alexa 647 and Alexa 750 data sets was carried out in three steps. First, the Alexa 647 image was warped using a polynomial warp transform, which corrects for differences in rotation, magnification, and higher order distortions between the two detection channels (the warping function was calculated based on previously obtained images of fiducial markers visible in both channels). Next, the Alexa 647 and Alexa 750 channels were independently drift corrected to account for lateral sample drift during data acquisition. Finally, the Alexa 647 and Alexa 750 images were registered by aligning the centroids of the clusters of localizations corresponding to fiducial markers present in each channel. This final registration step was a simple lateral translation. Scale bar 2 micrometers. (**B, C**) Magnified views of the boxed regions from panel A, showing the fully registered tubulin images from the Alexa 647 and Alexa 750 channels. Scale bars 250 nm. (**D**) A histogram showing the residual alignment error calculated for the 15 fiducial marks used in this example. The average registration error is less than 6 nm across the full image area.

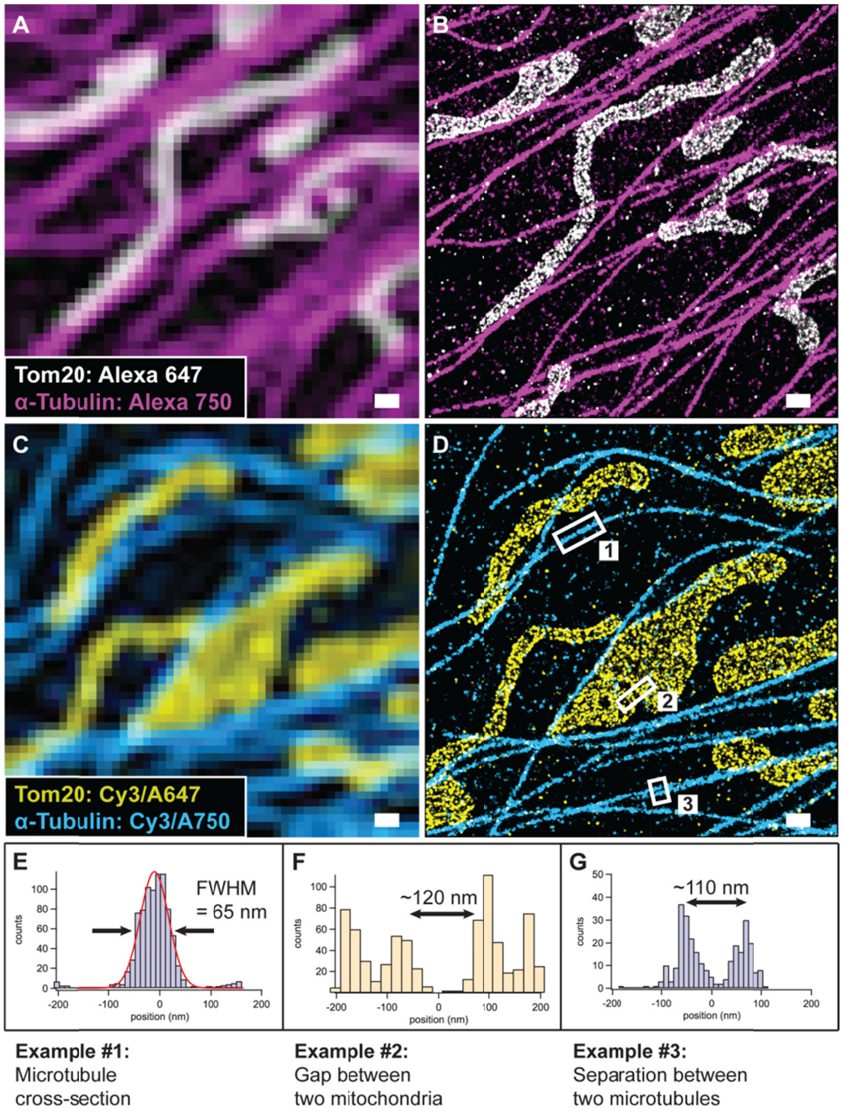

Figure S5: Image detail in dual emission channel STORM data. (**A**) Conventional fluorescence image of microtubules (magenta) and mitochondria (white) and (**B**) the corresponding STORM image from Figure 2A. The sample was labeled with Alexa 750 (for tubulin) and Alexa 647 (for Tom20). (**C**) Conventional fluorescence image of microtubules (cyan) and mitochondria (yellow) and (**D**) the corresponding STORM image from Figure 2D. The sample was labeled with Cy3-Alexa 750 (for tubulin) and Cy3-Alexa 647 (for Tom20). (**E**) Width distribution of localizations for the segment of the microtubule in box 1 in panel D. The measured 65 nm width of microtubule is wider than the true microtubule width due to the two-tiers of antibodies used to label the microtubules and the finite localization precision for the fluorophore. The separation between (**F**) two mitochondria (~120 nm) and (**G**) two microtubules (~110 nm), is well-below the diffraction-limited spatial resolution, data taken from boxes 2 and 3, respectively, in panel D. All scale bars 500 nm.

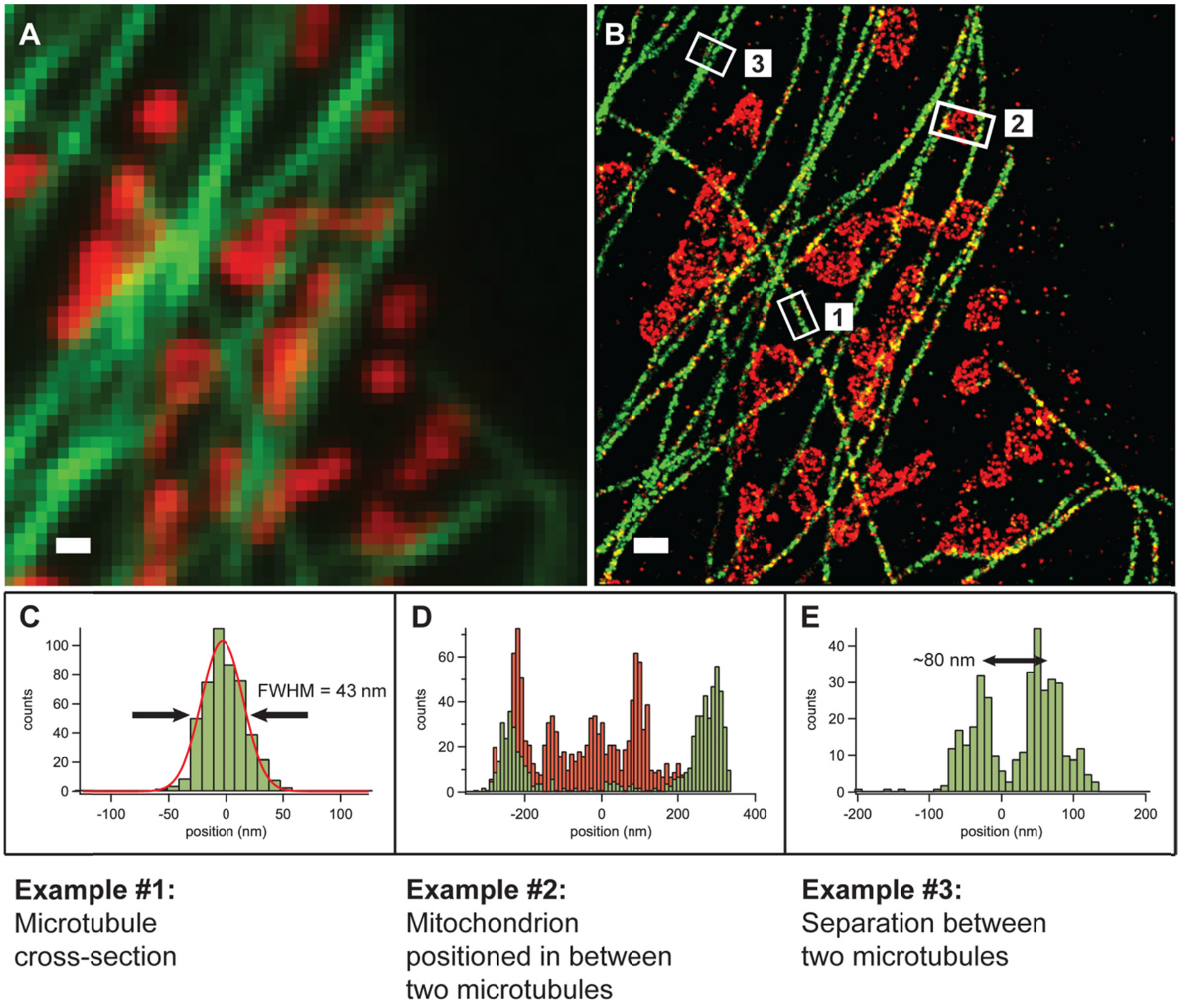

Figure S6: Image detail in dual activation channel STORM data. The sample was labeled with Alexa 405-Alexa 647 (for tubulin) and Cy3-Alexa 647 (for Tom20). (**A**) Conventional fluorescence image of microtubules (green) and mitochondria (red) and (**B**) the corresponding STORM image from Figure 2G. (**C**) The width distribution of localizations for the segment of the microtubule in box 1 in panel B. The reduced width of 43 nm compared to that of Alexa 750 shown in Figure 4E is due to the improved localization precision for Alexa 647. (**D**) A mitochondrion between two microtubules is easily resolved, as evidenced by the localization distribution from box 2 in panel B. (**E**) The separation between two microtubules (~80 nm) is well below the diffraction-limited spatial resolution, data from box 3 in panel B. All scale bars 500 nm.

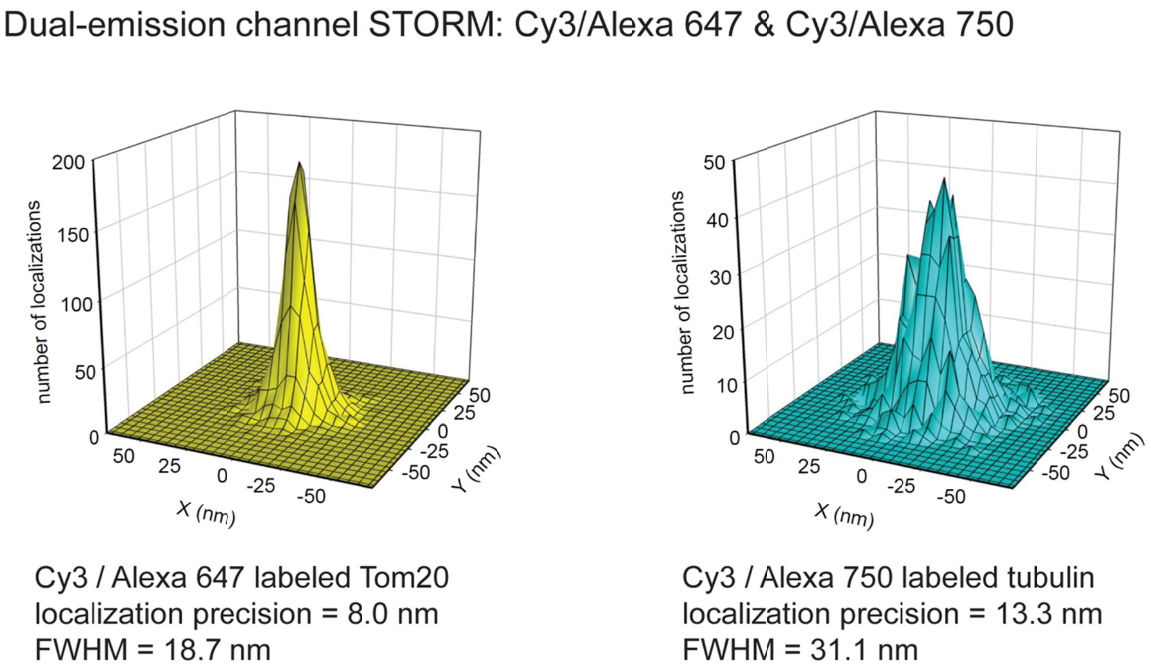

Figure S7: Localization precision of Alexa 750 and Alexa 647. Isolated clusters of localizations from Figure 2D – F were aligned according to their center of mass and a histogram of the spatial distribution of localizations was generated. The distributions were fit with a two-dimensional Gaussian function to determine the standard deviation, corresponding to the localization precision; and full-width at half-maximum, which corresponds to the minimum resolvable distance between two fluorophores in the image.

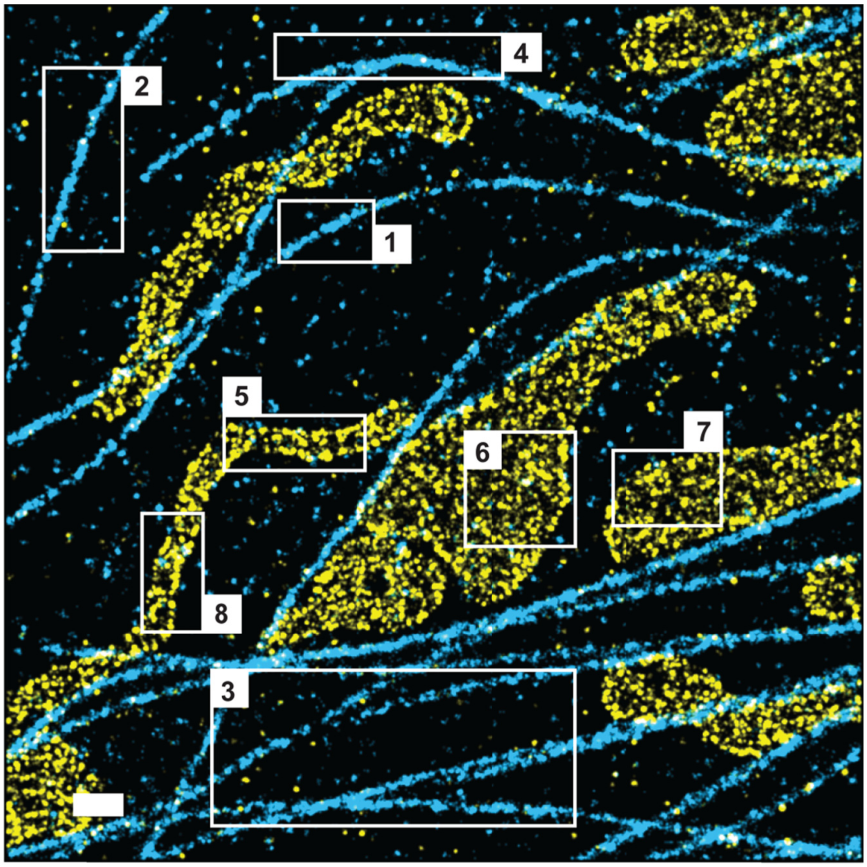

	Region #	Fraction Tom20 localizations	Fraction tubulin localizations
Tubulin regions	1	0.01	0.99
	2	0.08	0.92
	3	0.08	0.92
	4	0.05	0.95
Tom20 regions	5	0.95	0.05
	6	0.95	0.05
	7	0.96	0.04
	8	0.93	0.07

Figure S8: Crosstalk analysis for the dual emission channel STORM data. The table below the image shows the fraction of localizations identified from each of the two channels within the labeled boxes. Boxed regions 1 through 4 correspond to parts of the image with no apparent mitochondrial structure. Consistent with this, the quantitative analysis shows that >92% of the localizations are correctly assigned to the microtubule channel in each case. Similarly, for boxed regions 5 through 8, where the crosstalk of isolated mitochondria are analyzed, >93% of the localizations are assigned to the correct channel. Scale bar 500 nm.

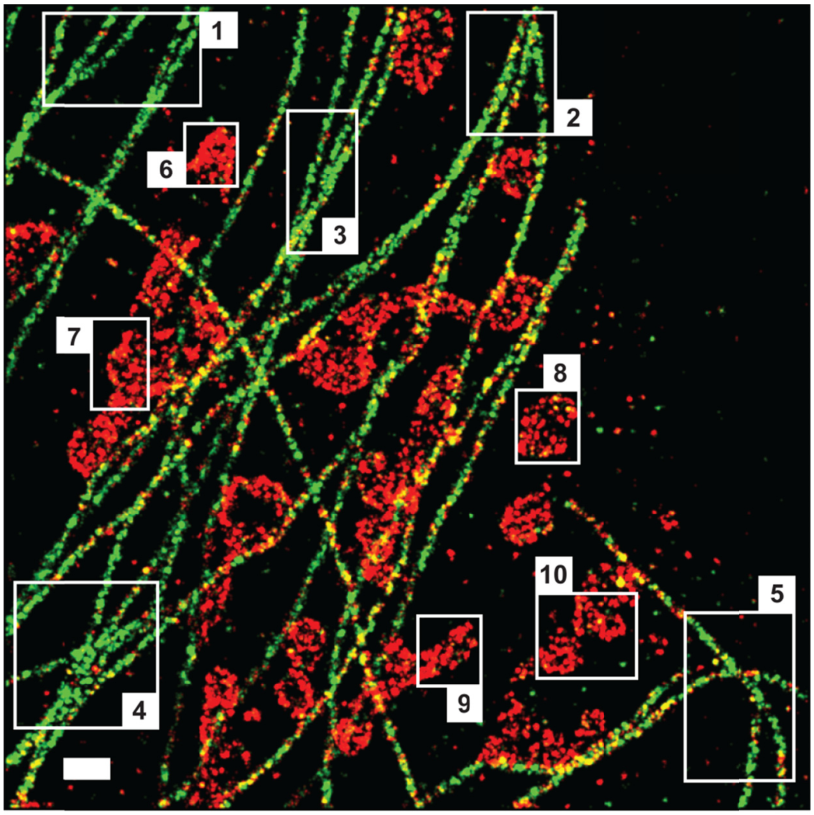

	Region #	Before crosstalk correction		After crosstalk correction	
		Fraction Tom20 localizations	Fraction tubulin localizations	Fraction Tom20 localizations	Fraction tubulin localizations
Tubulin regions	1	0.12	0.88	0.03	0.97
	2	0.23	0.77	0.13	0.87
	3	0.19	0.81	0.09	0.91
	4	0.17	0.83	0.08	0.92
	5	0.25	0.75	0.18	0.82
Tom20 regions	6	0.86	0.14	0.95	0.05
	7	0.84	0.16	0.92	0.08
	8	0.85	0.15	0.90	0.10
	9	0.88	0.12	0.94	0.06
	10	0.89	0.11	0.94	0.06

Figure S9: Crosstalk analysis for the dual activation channel STORM data. The table below the image shows the fraction of localizations identified from each of the two channels within the labeled boxes, before and after computational crosstalk correction. Boxed regions 1 through 5 correspond to parts of the image with no apparent mitochondrial structure. From the quantitative analysis, >75% of the localizations are correctly assigned to the microtubule channel before subtraction, which improves to >82% after correction. Similarly, for boxed regions 6 through 10, where isolated mitochondria were analyzed, the correct channel assignment improves from >84% to >90% after crosstalk correction. Scale bar 500 nm.

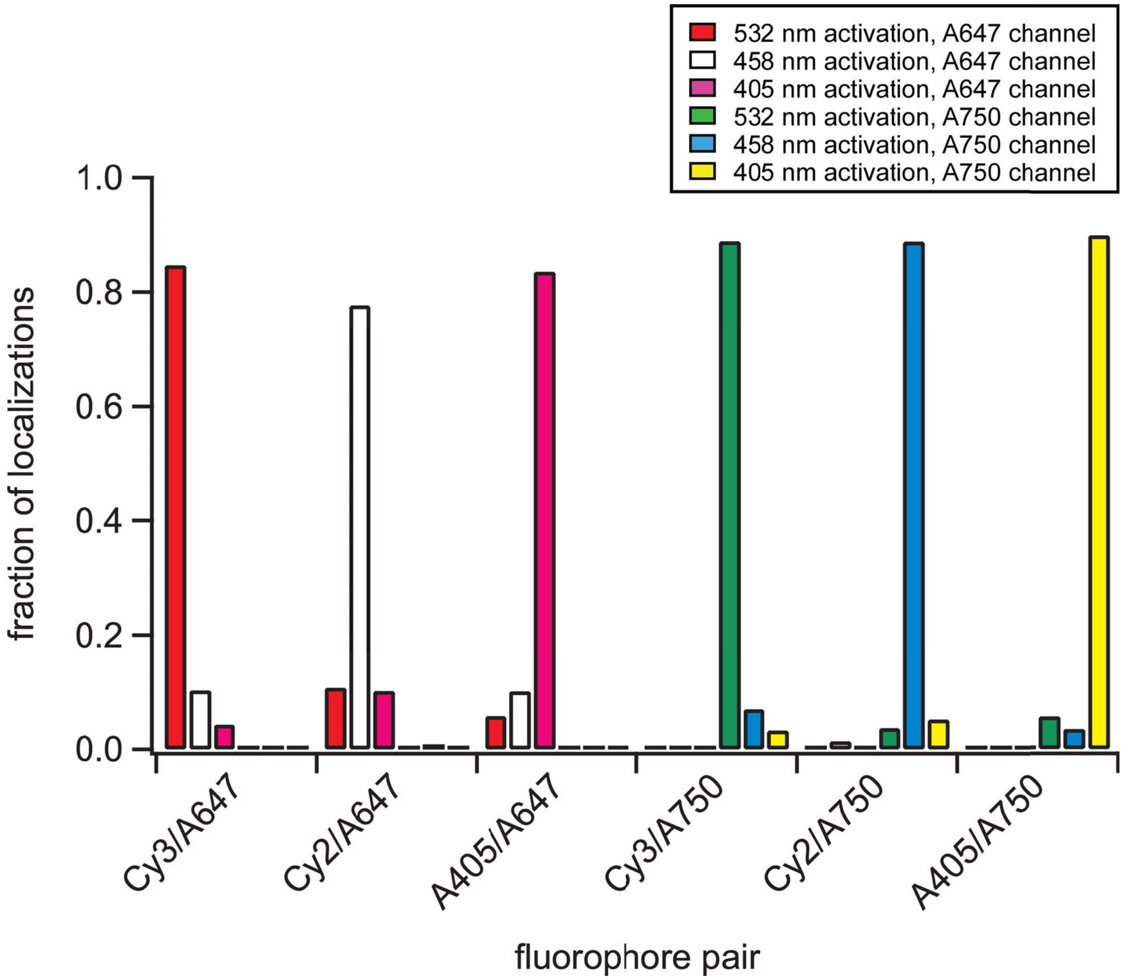

Figure S10: Crosstalk analysis for the six-color STORM image shown in Figure 3. Clusters of localizations within the six-color STORM image were selected for each fluorophore pair, and the fraction of localizations coming from each of the six probes was determined. The bar plot shows that in each case, correct channel assignment occurred ~80-90% of the time (e.g. >80% of localizations within Cy3-Alexa 647 clusters were correctly identified as being detected after 532 nm activation and in the Alexa 647 channel, red bar).

References

[1] M. Bates, B. Huang, G. T. Dempsey, X. Zhuang, *Science* **2007**, *317*, 1749-1753.
[2] B. Huang, W. Wang, M. Bates, X. Zhuang, *Science* **2008**, *319*, 810-813.
[3] B. Huang, S. A. Jones, B. Brandenburg, X. Zhuang, *Nat Meth* **2008**, *5*, 1047-1052.
[4] A. Dani, B. Huang, J. Bergan, C. Dulac, X. Zhuang, *Neuron* **2010**, *68*, 843-856.
[5] W. Leung, C. Cheung, S. Yue, Life Technologies Corporation, United States, **2010**.
